# Do Porpoises Choose Their Associates? A New Method for Analyzing Social Relationships among Cetaceans

**DOI:** 10.1371/journal.pone.0028836

**Published:** 2011-12-21

**Authors:** Mai Sakai, Ding Wang, Kexiong Wang, Songhai Li, Tomonari Akamatsu

**Affiliations:** 1 Center for International Cooperation, Ocean Research Institute, The University of Tokyo, Tokyo, Japan; 2 Life Science Network, The University of Tokyo, Tokyo, Japan; 3 Key Laboratory of Aquatic Biodiversity and Conservation of the Chinese Academy of Sciences, Institute of Hydrobiology of the Chinese Academy of Sciences, Wuhan, China; 4 National Research Institute of Fisheries Engineering, Fisheries Research Agency, Ibaraki, Japan; Institut Pluridisciplinaire Hubert Curien, France

## Abstract

**Background:**

Observing and monitoring the underwater social interactions of cetaceans is challenging. Therefore, previous cetacean studies have monitored these interactions by surface observations. However, because cetaceans spend most of their time underwater, it is important that their underwater behavior is also continuously monitored to better understand their social relationships and social structure. The finless porpoise is small and has no dorsal fin. It is difficult to observe this species in the wild, and little is known of its sociality.

**Methodology/Principal Findings:**

The swim depths of 6 free-ranging finless porpoises were simultaneously recorded using a time-synchronized bio-logging system. Synchronous diving was used as an index of association. Two pairs, #27 (an immature female estimated to be 3.5 years old) and #32 (an adult male), #28 (a juvenile male estimated to be 2 years old) and #29 (an adult male), tended to participate in long periods of synchronized diving more frequently than 13 other possible pairs, indicating that the 4 porpoises chose their social partners. The adult males (#32, #29) tended to follow the immature female (#27) and juvenile male (#28), respectively. However, during synchronized diving, the role of an initiator often changed within the pair, and their body movements appeared to be non-agonistic, e.g., rubbing of bodies against one another instead of that on one-side, as observed with chasing and escaping behaviors.

**Conclusions/Significance:**

The present study employed a time-synchronized bio-logging method to observe the social relationships of free-ranging aquatic animals based on swimming depth. The results suggest that certain individuals form associations even if they are not a mother and calf pair. Long synchronized dives occurred when particular members were reunited, and this suggests that the synchronized dives were not a by-product of opportunistic aggregation.

## Introduction

To understand the social relationships and social structure of animal communities, it is important to investigate the participants, initiators, and recipients of social interactions [1]. The fundamental elements involved when examining animal social structure are behavioral interactions that occur between two or sometimes more individuals [2].

Cetaceans have various social structures, including matrilineal groups (the killer whale, *Orcinus orca* [3]); longitudinal alliances of males (bottlenose dolphins, *Tursiops* sp. [4]); or pairs mainly consisting of mothers and calves (harbor porpoises, *Phocaena phocaena* [5]). Observing and monitoring the underwater behavioral interactions of cetaceans is challenging, especially in the wild. Based on spatial ranges, behavior types, and other data acquired by surface observation, previous cetacean studies have defined “associations” as circumstances in which interactions occur between individuals [6]. In general, such studies have utilized photo-identification methods using various platforms such as boats, airplanes, or land-based stations. Very few attempts have been made to monitor underwater behavior and analyze the initiators and recipients of social interactions among wild cetaceans [7], [8]. Because cetaceans spend most of their time underwater, it is important that their underwater behavior is also continuously monitored to further understand their social relationships and social structure.

The finless porpoise (*Neophocaena phocaenoides* G. Cuvier, 1829) is a small cetacean with a range that extends throughout the coastal waters of Asia and the freshwater system of the Yangtze River in China. The species is small (adult body length is approximately 1.5 m), swims quietly, and has no dorsal fin [9]. Consequently, it is difficult to observe and monitor this species in the wild using photo-identification techniques. Till date, little is known about the sociality of this species in the wild. Previous studies have concluded that the social structure of this species appears to be undeveloped and that the mother–calf pair is probably the only stable social unit [10].

In this study, we simultaneously monitored the diving behavior of 6 finless porpoises under free-ranging conditions using time-synchronized bio-logging systems. By defining synchronous diving as an index of “association,” i.e., circumstances in which individuals interact, we could examine the underwater associations of the porpoises and their time-sequential initiating and following behaviors. We tested whether the animals chose their associates and whether they were unilateral initiators or followers or swapped roles during synchronous diving.

**Figure 1:**
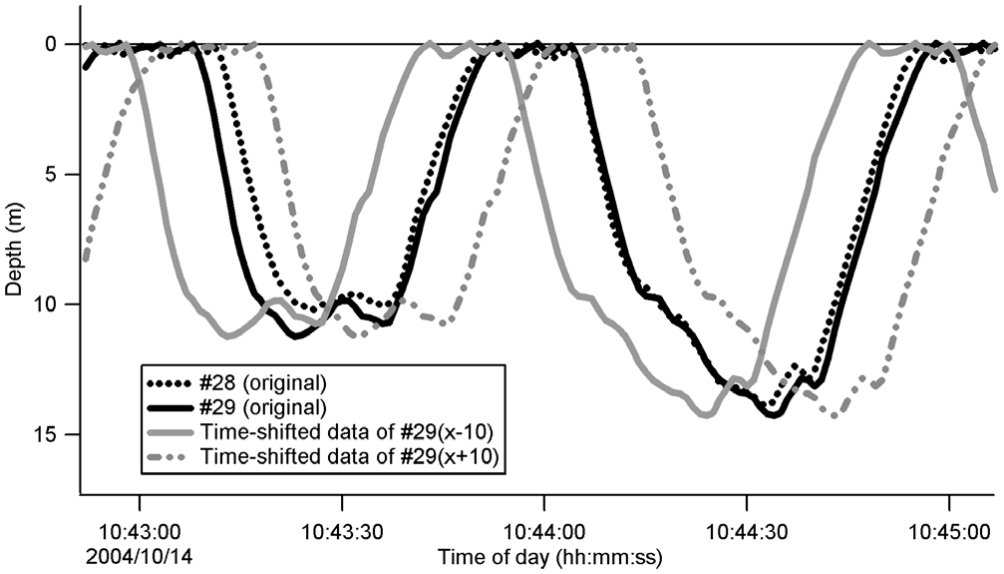
Example of time-shifted data for the swimming depth of a partner. Individual #28 was the reference individual and individual #29 was the partner.

Our hypothesis was that porpoises synchronize their swimming depth with that of their partner (true synchronous dives). To test this hypothesis, a precise definition of synchronous dive (hereafter referred to as SD) was required. The “reference individual” in the present paper refers to the individual whose dive profile was used as a reference dataset. We compared the time-shifted depth profiles of other individuals with the depth profile of the reference individual to identify associations. Time-shifted data were obtained by shifting the depth profile of a partner forward or backward in time by up to 10 s (Figure 1). We then calculated the depth differences between the reference and time-shifted data points for the possible partner. SD periods when the depth difference was within 1.5 m were added together to calculate the total SD duration. The long total SD duration was used to determine associations between individuals. The time-shifting period that maximized the total SD duration was used as the behavioral time lag for identifying the initiator and follower. To measure this lag, we needed to precisely synchronize the clocks of the data loggers (details can be found in the Appendix S1). A positive shift suggested that the partner was the follower, while a negative shift suggested that the partner was the initiator. If the SD period occurred coincidentally, the summation values would not be high, even with time shifting of the data. In this case, the pair was not defined as an association. All 15 possible pairs were examined. Note that the swimming depth of the partner could be <1.5 m in cases where the reference individual swam at a depth of 1.5–3.0 m, and the depth difference between the reference individual and partner was <1.5 m. Such cases were included in the analysis.

A short SD length would be more likely than a long SD length when the synchronization occurred coincidentally. A statistically objective broken-stick model was used for all combinations of SD length for the 15 possible pairs to split the SD length into 2 groups. The group with shorter SD lengths was considered to display coincidental SD and was excluded from further analysis. The group with longer SD lengths was considered to reflect true synchronization.

The second hypothesis tested was that a partner actively chose an associate, rather than randomly choosing a swimming mate. To test this hypothesis, the number of long SDs for each reference individual and 5 partners were compared using chi-square tests. Bonferroni corrections were applied to P values, with significance set at P<0.005. If a mate preference existed, a long SD would occur significantly more frequently with a specific partner than with other partners.

**Figure 2:**
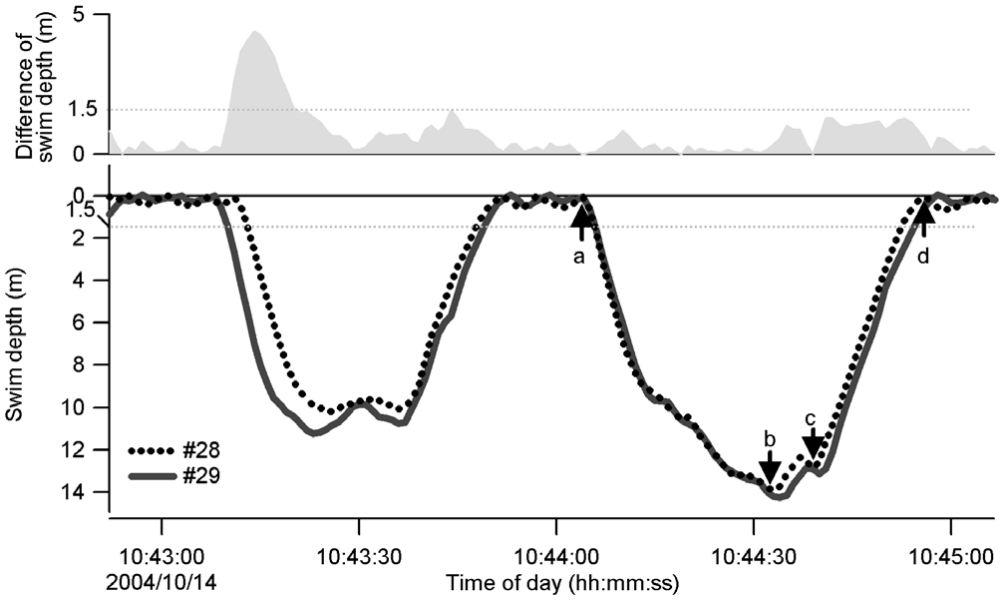
An example of synchronous diving in 2 finless porpoises. The first dive was not considered a synchronous dive (SD), whereas the second was considered SD. The initiator and follower were determined at 4 points: starting a dive (a), reaching a maximum depth (b), starting an ascent (c), and ending the dive (d).

The third hypothesis was that of an unequal partnership in which 1 porpoise in a pair tends to lead the other during synchronized swimming. To test the roles of an initiator and a follower within a pair during long SDs, the time points of 4 dive events, (a) starting a dive, (b) reaching a maximum depth, (c) starting an ascent, and (d) ending the dive, were measured for both individuals and compared within the pair (Figure 2). The individual that was ahead at the time of each event was considered to be the initiator, while the individual that was behind was considered to be the follower. For each individual, a chi-square test was used to compare the number of cases in which the individual was the initiator or follower, with significance set at P<0.05. As mentioned above, the time-shifting period that maximized the total SD duration provided the time lag for the initiator and follower was also used for testing this hypothesis.

## Results

### Synchronized swimming of pairs

**Figure 3:**
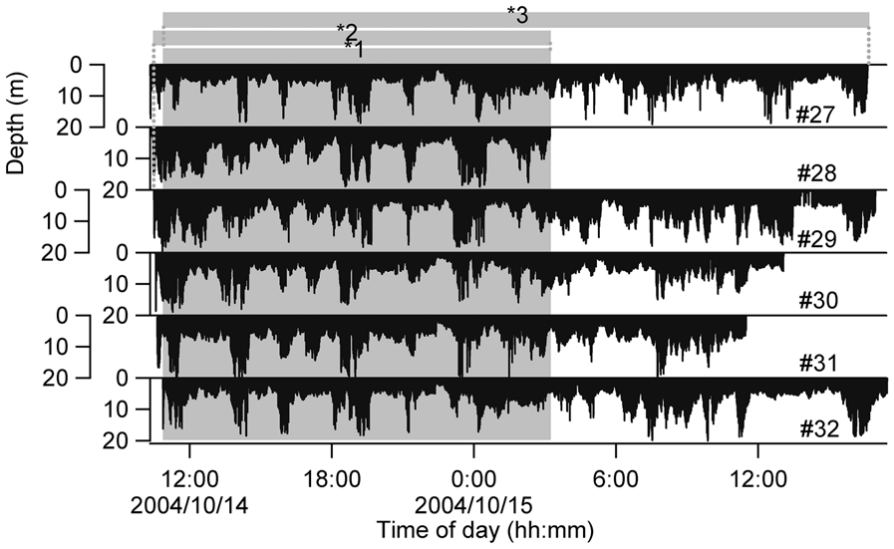
Overview of the dive profiles of the 6 finless porpoises. *1: the dataset used for testing the presence of true synchronized dives and testing partner choice by the 6 porpoises (16.4 h). *2: the dataset used for determining the identity of the initiator and follower for individuals #28 and #29 (16.7 h). *3: the dataset used for determining the identity of the initiator and follower for individuals #27 and #32 (29.7 h).

**Figure 4:**
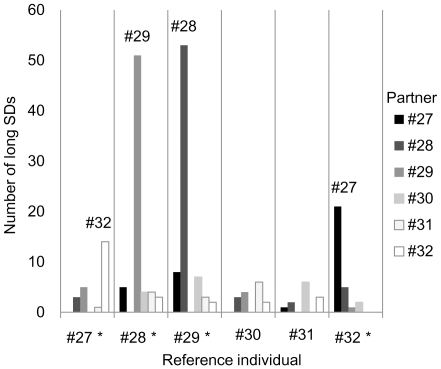
Distribution of synchronous dive (SD) durations. The figures in the upper left of each graph indicate the reference individual ID number. The points with a time lag of 0 show the SD duration as calculated from the original data of the reference individual and those of the partners. The points with a time lag of −10 s to −1 s and 1 s to 10 s show the SD duration as calculated from the original data of the reference individual and the time-shifted data of the partners, which was the original data staggered from −10 s to 10 s, respectively.

In total, 159.6 h of swimming depth data were collected from 6 porpoises. Table 1 and Figure 3 show an overview of the dive profiles of these 6 animals. A total of 16.4 h of simultaneous recordings of all 6 animals was collected and used for testing the abovementioned first and second hypotheses. The number of possible pairings among the 6 individuals is 15. Figure 4 presents the total SD duration among all pairs based on the length of the time shift. When individual #27 was the reference individual, the total SD duration with individual #32 was longer than that with other individuals (Figure 4A). Similarly, when individual #32 was the reference individual, individual #27 was the partner with the longest SD duration (Figure 4F). When individual #28 was the reference individual, the total SD duration with individual #29 was longer than that with other individuals (Figure 4B), and when individual #29 was the reference individual, individual #28 was the partner with the longest SD duration (Figure 4C). On the other hand, individuals #30 and #31 did not show any peak SD durations, which indicates that no synchronized diving occurred with any of the other animals investigated (Figure 4D, Figure 4E). These results indicate that the 2 pairs (i.e., #27 and #32, #28 and #29) frequently dove synchronously. SDs with more than 2 porpoises were not recorded.

**Table 1:** Information relating to the 6 Yangtze finless porpoises to which data loggers were attached.

ID	Age class & sex	Body length (cm)	Body weight (kg)	Estimated age	Dive duration* (s): Avg. ± S.D., Max
#27	Immature female	131.0	39.4	3.5 years	43±38, 162
#28	Juvenile male	123.0	34.0	2 years	32±27, 134
#29	Adult male	148.5	48.7	unknown	24±20, 107
#30	Adult male	159.0	59.4	unknown	39±34, 135
#31	Adult male	146.5	48.5	unknown	34±31, 141
#32	Adult male	147.0	43.5	unknown	30±28, 120

*; calculated using data from 16.4 h of simultaneous recordings for all 6 animals.

The total SD duration in which individual #27 was the reference individual and individual #32 was the partner was 1421 s. This duration was 2053 s with individual #32 as the reference individual and individual #27 as the partner (Figure 4A, Figure 4F). Asymmetric durations were caused by the decision to exclude shallow swimming durations of the partner during data screening. The depth difference between the reference individual and partner was calculated when the reference individual swam deeper than 1.5 m, even if the partner swam at a shallower depth. In this case, SD duration was counted for the reference individual but not for the partner. Asymmetric durations were also caused by dives when the depth differences were maintained within 1.5 m from the start to the end of the dive for the reference individual but not for the partner. For example, if the partner continued its dive after the end of the dive for the reference individual. In this case, SD duration was counted for the reference individual but not for the partner. In the #27 and #32 pair, individual #27 tended to swim at shallower depths and/or for longer duration than individual #32, which caused shorter SD durations when individual #27 was the reference individual. Similarly, the total SD duration was 2958 s when individual #28 was the reference individual and individual #29 was the partner. It was 3151 s for the reverse pair (individual #29 as the reference individual and individual #28 as the partner; Figure 4B, Figure 4C).

**Figure 5:**
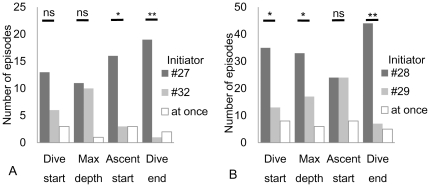
Log frequency for evaluating synchronous dives (SD) that occurred coincidentally. Logarithms of actual frequencies of SD length were plotted against SD lengths and approximated to a broken-stick curve. Black dots represent the group with shorter SD lengths (<21 s), and white dots represent the group with longer SD lengths (>21 s).

SD durations ranged from 1–99 s during 16.4 h of simultaneous recordings of 6 animals (Figure 3). To split the SD length into 2 groups, a broken-stick model was used for all samples of SD length for the 15 pairs. The residual sum of squares was smallest when the samples were divided into 2 groups, one with SD durations of 1–21 s and one with SD durations >21 s (Figure 5). The group with shorter durations was considered to be coincidentally swimming synchronously, and the group with longer durations was considered to mainly include true SDs.

### Partner preference

**Figure 6:**
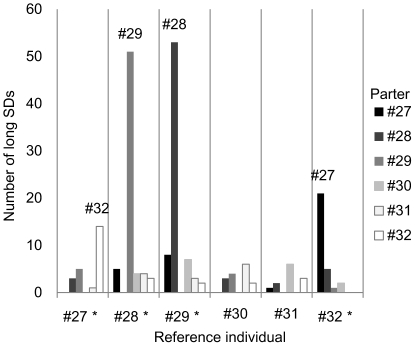
Comparison of the number of long synchronous dives (SDs) performed with their partners by the 6 porpoises. The partners for 4 porpoises were not uniformly distributed (*: 1×5 chi-square test, P<0.001).

Only long SD durations (>21 s) were used for this analysis. The number of long SDs with partners was compared for each reference individual (Figure 6).

The number of long SDs for the 4 reference porpoises (#27, #28, #29, and #32) with 5 partners was not uniformly distributed (1×5 chi-square test, P<0.001). When individual #27 was the reference individual, the number of long SDs with individual #32 was significantly higher than that with individuals #30 and #31(chi-square test, Bonferroni correction, P>0.005). Analysis of the reverse pair, #32–#27, also showed significantly more frequent longer SDs than with all 4 other pairs (chi-square test, Bonferroni correction, P>0.005). Similarly, the #28 and #29 pair showed long SDs significantly more frequently than all 4 other pairs (chi-square test, Bonferroni correction, P>0.005). These results suggest that the 2 pairs, #27 and #32, #28 and #29, more frequently swam together than with other individuals.

### Do particular porpoises play the initiator?

The SD durations showed a mirror-symmetric distribution between individuals in the 2 pairs. The total SD duration of the #27–#32 pair was dependent on time shifting. SDs showed local maxima at +1 s and +6 s for this pair, and SDs of the #32–#27 pair showed local maxima at −2 s and −6 s (Figure 4A, Figure 4F). This indicates that porpoise #32 tended to follow porpoise #27 after 1–2 s or 6 s. The total SD duration of the #28–#29 pair was maximum at +1 s, and the total SD duration of the reverse pair was maximum at −1 s (Figure 4B, Figure 4C). This indicates that porpoise #29 tended to follow porpoise #28 after 1 s.

**Figure 7:**
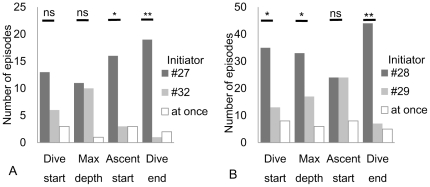
Number of episodes of being an initiator or a follower for each individual at 4 points in a dive bout. Binominal test, two-tailed; ns, not significant; *: P<0.05; **: P<0.001. “At once” means that 2 individuals passed the predefined event simultaneously.

Initiator and follower relationships were manually examined by comparing the dive depth during the long SDs of the pairs (#27–#32 and #28–#29). In total, 22 long SD events (maximum 100 s; average ± standard deviation, 51±23 s) were identified with individual #27 as the reference individual and individual #32 as the partner during 29.7 h of simultaneous recordings (Figure 3). For the pair with individual #28 as the reference individual and individual #29 as the partner, 56 long SDs (86 s, 42±15 s) were identified during 16.7 h of recordings (Figure 3). In the #27–#32 pair, long SDs occurred from 1400 to 1500, 1700 to 1800, and 2100 to 2400 h on October 14 and 0500 to 0600 h, and 1400 to 1600 h on October 15. In the #28–#29 pair, long SDs occurred from 1000 to 1800 h and 2000 to 2100 h on October 14 and 0000 to 0200 h on October 15. Individual #27 tended to start to ascend and end diving earlier than individual #32 (Figure 7A). Individual #28 tended to start diving, reach a maximum depth, and end diving earlier than individual #29 (Figure 7B). There were initiating individuals (#28 and #27) in the pairs in these 78 long SDs. Instances where the identity of the initiator changed during a long SD (N = 60) were more frequent than those where 1 porpoise consistently acted as the initiator (N = 18). As shown in Figure 7, the 2 porpoises often passed a predefined dive event simultaneously.

**Figure 8:**
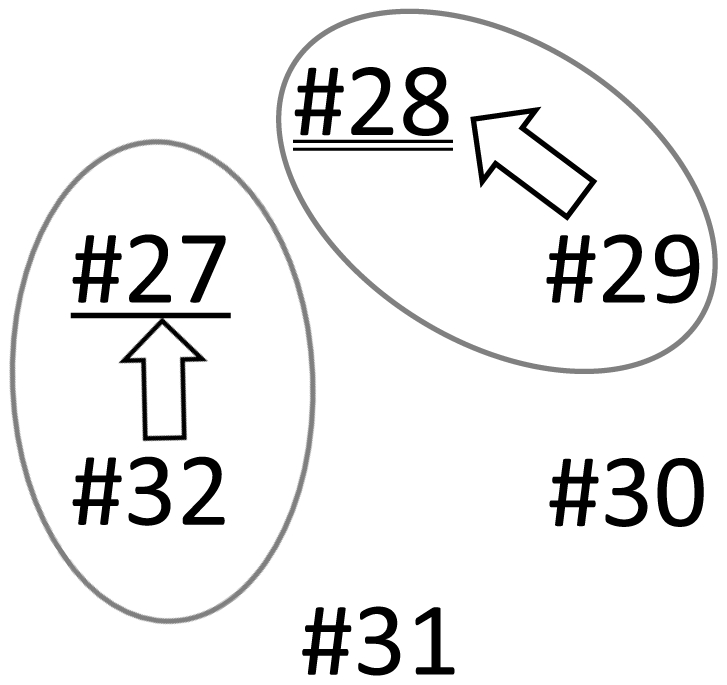
Social relationships among the 6 porpoises as revealed by this study. The underlined number indicates an immature female, and the double underlined number indicates a juvenile male. The others are adult males. The tips of the arrows indicate the initiators, and the bases of the arrows indicate the followers.

Figure 8 shows the social relationships of the 6 porpoises, as revealed by this study. The 2 pairs, #27 (immature female) and #32 (adult male), #28 (juvenile male) and #29 (adult male), frequently tended to maintain long synchronized diving. The adult males (#32, #29) tended to follow the immature female (#27) and juvenile male (#28) during synchronized diving, respectively.

## Discussion

Our study demonstrated that time-synchronized depth data could detect synchronous diving among free-ranging porpoises. Maintaining a close swimming distance throughout dive bouts suggests that porpoises have specific preferences for a partner and initiator and follower roles. These SDs can be regarded as associations, i.e., circumstances in which interactions usually occur between animals [6].

The total SD duration for each pair (Figure 4) included short SDs that occurred coincidentally. Therefore, data of naïve analysis using the dive profile data of aquatic animals could be contaminated with random associations that occur coincidentally. Our method allowed for the identification and removal of random associations from the SD data for each pair and for testing whether specific pairs associated significantly more frequently than other possible pairs. Two pairs, #27 (immature female) and #32 (adult male), #28 (juvenile male) and #29 (adult male), swam together significantly more often than the other 13 possible pairs that could be formed by these 6 porpoises. Other porpoises that did not carrying data loggers were also possibly part of these associations, although we did not monitor any other untagged individuals in this study.

Previous studies of finless porpoises have concluded that the social structure of this species seems to be undeveloped and that mother–calf pairs were probably the only stable social unit [10]. However, our results suggest that particular pairs form associations, even if they are not mother and calf pairs. Long SDs occurred intermittently and repeatedly throughout the day. This suggests that long SDs occurred when particular members were reunited and were not by-products of opportunistic aggregation.

This is the first report of a finless porpoise pair consisting of an immature female (#27) and an adult male (#32). Sexual maturation takes place at age 4–6 years in this species [9]. Individual #27 was estimated to be 3.5 years old and would possibly have been mature several months later. The breeding season of Yangtze finless porpoises is believed to be between March and September [11], [12]. Male Dall's porpoises (*Phocoenoides dalli*) reportedly have a tendency to associate closely with females for mate guarding during the breeding season [13]. It is possible that male and female finless porpoises formed an association and that the male was guarding a potential mating partner for the next breeding season. Individual #28 was an immature 2-year-old male that may already have been weaned because the nursing period in finless porpoises is considered to last 7 months [9]. Associations of multiple mature males have been reported for several mammal species including dolphins (bottlenose dolphins [4]), lions (*Panthera leo*) [14], and chimpanzees (*Pan troglodytes*) [15]. Subadult males also associate with peers (bottlenose dolphins [4], sperm whales (*Physeter macrocephalus*) [16], waterbuck (*Kobus ellipsiprymnus*) [17]). However, associations of adult and juvenile males, as found in this study, occur very rarely among mammals, one exception being the banded mongoose (*Mungos mungo*) [18].We could not determine the function of SDs or whether other behaviors were shown during SDs of the porpoises. Synchronous diving in penguins has been suggested to increase foraging efficiency [19] and predator avoidance [20]. The porpoises in this study do not need to avoid predators as they are in a reserve. The relationship between SDs and foraging in finless porpoises is uncertain. When porpoises search and try to capture prey, body rolling and speed dropping occurred [21]. Analyses of body angle and swim speed during SDs in further studies should reveal the occurrence of the synchronous foraging behavior.

The total SD duration with the time-shifted data of the partner was longer than that with the original data of the partner (Figure 4A–4C and Figure 4F). This indicates that porpoises tended to be initiators or followers. In this study, an adult male (#32) tended to follow an immature female (#27), and another adult male (#29) tended to follow a juvenile male (#28). The timing of the start and end of the dive was determined by the initiator. The follower might continue SD by referring to the movement of the initiator. The smaller animals (#27 and #28) tended to be the initiators. This might be linked to the different physiological abilities associated with the different body sizes, e.g., larger animals are able to dive for longer periods. However, individuals #27 and #28 tended to dive longer than their partners during this study (Table 1). Furthermore, the porpoises studied would not need long dive close to their physiological limit because the environment is shallow (<20 m). Therefore, the critical factor for deciding to be an initiator or a follower might have been based on social context rather than on any the difference in physical capacity.

Exchange of the initiator role within pairs occurred frequently during a single dive, and 2 porpoises often passed a predefined dive event, such as reaching a maximum depth, simultaneously (Figure 7). This suggests that the behavior during SDs was not one-sided (e.g., chasing and escaping). Non-agonistic social behaviors have been observed in captive finless porpoises. For example, in captive Yangtze finless porpoises, sociosexual behavior [22], [23] and rubbing of the body against a ridge with horny tubercles have been observed (Sakai, unpublished). Moreover, a previous study reported that a female frequently approached a male and that this was followed by body contact or swimming in union [24]. Captive finless porpoises in Japan have also been observed to repeatedly rub against each other with their tubercles whilst overtaking each other (Noguchi, 2009, Masters Thesis, Tokyo Institute of Technology), and this behavior has been suggested to be indicative of affiliation. It is possible that social behavior occurred during SDs in this study.

It is uncertain how the porpoises maintained synchrony. Penguins have been suggested to maintain visual contact during synchronous diving [25], [26]. It would be difficult to follow a partner visually at our study site because of low water visibility (<1 m). The porpoises might thus have used an auditory or tactile (body contact or sensation of a water stream generated by the initiator) cue for maintaining synchrony.

In future, determining the function of SDs, identifying associated behaviors, and revealing the mechanism whereby synchrony is maintained will require analysis of body angles, tactile interactions, and sonar usage by porpoises during SDs using bio-logging methods, and these data will need to be matched with visual observations of captive finless porpoises.

## Materials and Methods

### Study site

In October 2004, data loggers (PD2GT; Little Leonardo, Tokyo, Japan) were attached to 6 finless porpoises (Table 1). The animals were captured from Tian-e-Zhou Oxbow (29°47′–29°51′N, 112°32′–112°37′E). After attaching the data loggers, the animals were released back into the oxbow, which is approximately 21 km long and 1–2 km wide. The oxbow has a maximum depth of approximately 20 m, and water visibility during the study period was <1 m. The oxbow forms part of the Tian-e-Zhou Baiji National Natural Reserve in Hubei Province, China. The reserve was established by the Chinese government in 1992 to protect the baiji (*Lipotes vexillifer*) and Yangtze finless porpoise (*N. phocaenoides asiaeorientalis*). Some porpoises were introduced from the main stream of the Yangtze River to this oxbow in 1990–2004, and they have been breeding in the oxbow naturally. The oxbow had 25 surviving animals during the study period in October 2004 [27]. The study site and the capture procedures have been described in detail in a previous study [28].

### Behavioral monitoring

A behavioral data logger (PD2GT; Little Leonardo, Tokyo, Japan) was attached to the left side of the body of each animal for recording depth, swimming speed relative to the water, and acceleration in the longitudinal and transverse axes. The sampling intervals for depth, speed, and acceleration were 1, 0.125, and 0.0625 s, respectively. Resolution of the depth sensor was 5 cm. We also attached an acoustic tag (A-tag) to the right side of the body of each animal [21]. Information from the acoustic tag could be used for time synchronization of the clock of each behavioral data logger among multiple animals. In this study, we did not analyze any biosonar behavior and only used the biosonar signal events as a time-synchronizing trigger (Refer to the Appendix S1 for details on the synchronization procedure).

Tags were attached to the body above the pectoral fin using a suction cup [21]. After spontaneously falling off, the suction cups together with the tags could be located and retrieved using VHF radio signals broadcasted by the tags (MM110; Advanced Telemetry Systems, Isanti, MN, USA).

### Ethics statement

This study did not include any human subjects or non-human primates, and thus did not require specific adherence to the Declaration of Helsinki or Weatherall report. The research was conducted under a permit issued by Hubei Fisheries Administration Bureau, Hubei Province, China. The approval number is EYUGUAN 2008-19.

### Data processing

Custom-made software on Igor Pro 6.0 (WaveMetrics, Lake Oswego, OR, USA) was used for data examination and processing. The porpoises typically repeated several respirations and short, shallow dives (approximately 0.4 m) for several seconds, followed by a long dive (Figure 2). Because the respiration phase occurred frequently, we had to exclude this phase to avoid counting synchronization that occurred coincidentally. Therefore, only dives deeper than 1.5 m were analyzed for the reference individual. A synchronous dive for a reference individual was defined as a dive in which the reference individual maintained its swim depth within a 1.5 m difference from the partner throughout the dive (Figure 2). The body length of an adult Yangtze finless porpoise is approximately 1.5 m, therefore, the pair may have engaged in social physical contact during synchronized dives, although horizontal distance was not determined. Even a short separation of >1.5 m disqualified the dive as a synchronized dive. This definition is conservative and probably excluded some synchronized dives in which animals did not maintain continuous close contacts. The beginning and end of a dive bout were defined as the time points when the animal swam across the 1.5 m layer separating it from the water surface during descent and ascent, respectively.

## Supporting Information

Appendix S1 **Time synchronization using acoustic bio-logging.**
(DOC)
