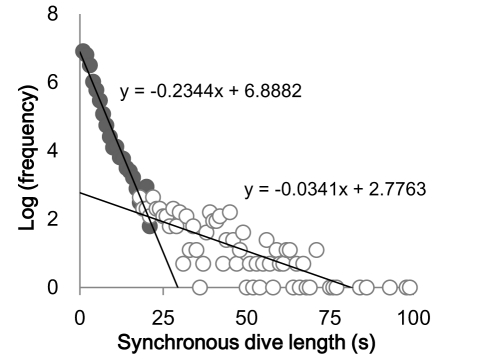# Correction: Do Porpoises Choose Their Associates? A New Method for Analyzing Social Relationships among Cetaceans

**DOI:** 10.1371/annotation/4529d94e-af8d-4ac5-b381-41323f8ece6c

**Published:** 2012-01-31

**Authors:** Mai Sakai, Ding Wang, Kexiong Wang, Songhai Li, Tomonari Akamatsu

There was an error in Figure 5. The correct Figure 5 can be viewed here: 

**Figure:**