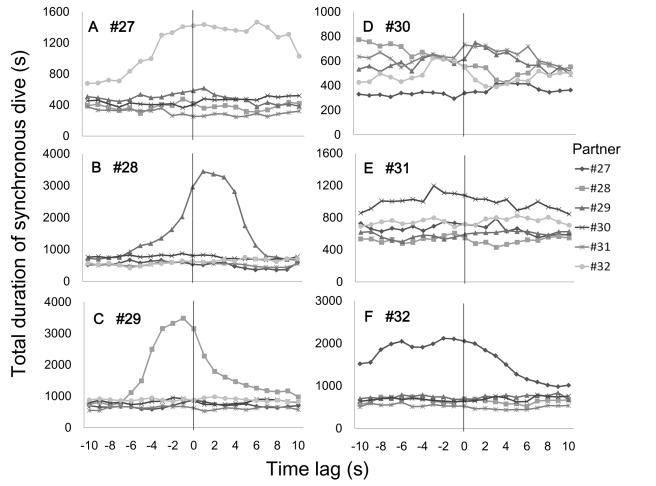# Correction: Do Porpoises Choose Their Associates? A New Method for Analyzing Social Relationships among Cetaceans

**DOI:** 10.1371/annotation/f7affdee-30fb-49c4-aeef-2710172d557e

**Published:** 2012-03-05

**Authors:** Mai Sakai, Ding Wang, Kexiong Wang, Songhai Li, Tomonari Akamatsu

There was an error in Figure 4. The correct Figure 4 can be viewed here: 

**Figure:**