# Supplementary material for: Do Porpoises Choose Their Associates? A New Method for Analyzing Social Relationships among Cetaceans
Source: PLoS One. 2011 Dec 21;6(12):e28836. doi: 10.1371/journal.pone.0028836 (PMC3244420; doi:10.1371/journal.pone.0028836)
Supplement: Appendix S1 — Time synchronization using acoustic bio-logging. (DOC) [file pone.0028836.s001.doc]

Time synchronization using acoustic bio-logging

We used acoustic tags to synchronize the clocks of the behavioral data loggers among multiple animals. The crystal clock employed in most bio-logging systems drifts 1 s within a couple of days, even after temperature calibration. The accumulated difference of each clock among bio-logging systems seriously affects observations of the group behavior of tagged animals. In the present study, respiration noise and vocalization signals were used to synchronize all behavior data loggers and acoustic tags.

The acoustic data logger consisted of 2 ultrasonic hydrophones (MHP-140ST; Marine Micro Technology, Saitama, Japan) with a band-pass filter (−3 dB with a 55–235 kHz range), a high gain amplifier (+60 dB), a central processing unit (PIC18F6620; Microchip, Detroit, MI, USA), flash memory (128 MB), and an off-the-shelf lithium battery (CR2), placed in an aluminum case that was pressure resistant to a depth of 200 m. A pulse event recorder (A-tag) stored the received sound pressure and time arrival difference between the 2 hydrophones every 0.5 ms, which is less than the minimum interclick interval of finless porpoises [25]. The details of this data logger have been described previously [24].

The clocks in the acoustic tags and behavioral data loggers were synchronized in advance and can drift by up to 2 s per day. To synchronize the 2 clocks present on a single animal, splash noises associated with respiration were used as signals for data matching. During respiration, porpoises tend to produce a splash, which creates broadband noise. At this moment, the propeller sensor is exposed to air and stopped. We compared the pattern of successive respirations recorded by both the acoustic tags and behavioral data loggers and removed clock drift and any initial offset.

To synchronize the clocks between the tags and data loggers on multiple animals, click pulse sequences recorded by multiple acoustic tags were used as signals. For example, if porpoise A emitted a click pulse sequence near porpoise B, data loggers on porpoises A and B recorded the same click pulse sequence. We compared these sequences and removed the clock drift between the data loggers on porpoises A and B. Thereafter, we removed the clock drift between the acoustic tag and behavioral data logger on each animal.
